# Effect of Covid-19 quarantine on diabetes Care in Children

**DOI:** 10.1186/s40842-021-00122-x

**Published:** 2021-05-21

**Authors:** Miriannette Gayoso, Whei Ying Lim, Madhuri S. Mulekar, Anne-Marie D. Kaulfers

**Affiliations:** 1 Department of Pediatrics, University of South Alabama, Strada Patient Care Center, 1601 Center St, Suite 1 S, Mobile, AL 36604 USA; 2 Department of Mathematics and Statistics, University of South Alabama, 411 N University Blvd North, Mobile, AL 36688 USA

**Keywords:** Diabetes, School nurse, COVID-19 quarantine

## Abstract

**Background:**

With the onset of the COVID-19 pandemic and state-mandated school closures in the spring of 2020, the management of type 1 diabetes in children underwent significant changes. The aim of our study was to assess the effect of stay-at-home orders on glycemic control in children.

**Methods:**

We conducted a retrospective review of 238 children with type 1 and type 2 diabetes who were seen in the Pediatric Endocrinology Clinic at the University of South Alabama. Average Hemoglobin A1c (A1c) levels in the year prior to stay-at home orders (May 2019April 2020) were compared with A1c values during the quarantine period (May 2020July 2020) using a paired t-test. We also analyzed the change of A1c level with respect to sex, race, type of diabetes, type of insurance, and mode of insulin administration, using a 2-sample t-test.

**Results:**

The average A1c significantly increased from 9.2% during the previous year to 9.5% during the quarantine period (*p*=0.0097). The increase of A1c was significantly higher in public insurance patients (0.49% increase) compared to private insurance patients (0.03% increase), (*p*=0.0137). We also observed a significant association between the direction of change and type of insurance. Forty-eight percent of public insurance patients had an A1c increase of >0.5% while 54% of private insurance patients had no change or decrease in A1c (*p*=0.0079).

**Conclusions:**

The COVID-19 pandemic resulted in worsening glycemic control in children with type 1 diabetes, with those on public insurance affected in greater proportion than those with private insurance.

## Background

Type 1 Diabetes is a chronic illness that requires constant attention and monitoring. Parents and school nurses play a critical role in helping these children maintain control of their diabetes [1]. If diabetes control is not regulated during the childhood years, then these children are at risk of developing complications later in life [2]. The severe acute respiratory syndrome coronavirus 2 (SARS-CoV-2, also called COVID-19) was introduced to The United States of America in early 2020, causing widespread illness [3]. Within a few months, state governors started issuing state-wide mandates to close schools, and asking everyone to stay at home. In Alabama, schools closed on March 18, 2020. For children with Type 1 Diabetes, this changed their daily routine, their eating habits, and also the primary caregiver. For some families, the school nurse previously provided most of the diabetes care, and now responsibility shifted back to the parent. For other families, the child was left to do the diabetes care on their own while the parent still went to work. This study was performed to evaluate the effect of the stay-at-home orders on glycemic control in children with Type 1 Diabetes.

## Methods

### Subjects

The retrospective cohort included all of the insulin-dependent diabetes patients seen at the Pediatric Endocrine clinic at the University of South Alabama in Mobile, Alabama, from May 1, 2020 to July 31, 2020. These dates were chosen in order to evaluate the change in Hemoglobin A1c (A1c) values after the stay-at-home orders, which started in mid-March 2020, about 1.5months before the start of our study. Even though A1c is a typically a 3month measure, it most closely correlates with the previous 812weeks [4]. From MayJuly 2020, we had 419 patients with type 1 or type 2 diabetes scheduled for a clinic visit. Thirty percent of these patients skipped or rescheduled their clinic visit, mostly due to COVID-19 concerns. In Alabama, COVID rates increased steadily from March-late July, then started declining again. No virtual visits were done at our institution during this time, because most of our patients were either unable to download their pump or sensor remotely, or did not have a pump or sensor. Of 316 patients seen in clinic, we excluded 78 patients from analysis: 48 were recently diagnosed, 25 were not on insulin, and 5 were new to this clinic. For 238 patients included in our study, we compared the A1c value obtained at their clinic visit with their own average A1c over the previous 12months (pre-quarantine one-year period). All of these patients had at least one A1c value measured in our clinic in the previous year. Of those, 95% patients had at least two A1c values, 69% had at least three, and 9% had four A1c values in the previous year. For each patient, the average of all the A1c values available over the pre-quarantine period was used for further analysis. Demographic data was collected on each patient from the medical records including sex, age, race, type of insurance (private vs public insurance/Medicaid), and mode of insulin administration (insulin pump vs multiple daily injections (MDI)). This study was approved by the Institutional Review Board at the University of South Alabama.

### Methods and measurements

All data collected was organized in an Excel spreadsheet without any identifying characteristics and was analyzed using statistical software JMP-Pro v 14.2.0 (A product of SAS Institute, Inc.). All categorical data was summarized using percentages and all numerical data was summarized using mean and standard deviation. Association between two categorical variables was studied using a Likelihood ratio Chi square test and difference in the averages of two groups was studied using a 2-sample t-test. The majority occurrence (i.e. >50%) of an event was studied using a one-proportion z-test and deviation from no change on the average outcome was studied using a one-sample t-test. Significance of the average change from the first to the second period was studied using a paired t-test. For race, the analysis was conducted using only Black vs white patients, because there were not enough patients in the Hispanic or Other Race category.

## Results

### Patient characteristics

Of the 238 study patients, 45% were female. Average age of patients in this study was 13.3years, with a range of 2 to 19years. Forty-five percent of patients were on a pump and the rest were on MDI. Twenty-nine percent were wearing a continuous glucose monitor. Sixty-one percent were white, 34% were Black, 3% were Hispanic, and the remaining 2% were of other races. Only 4% of patients had Type 2 Diabetes and were on insulin, while the other 96% had Type 1 Diabetes. Forty-seven percent were on public insurance/Medicaid (Alabama or Mississippi) and 53% were on private insurance, which includes Tri-Care (military insurance). See Table1 for details.

**Table 1:** Patient Characteristics

	N	% of Total
**Sex**		
Female	107	44.96%
Male	131	55.04%
**Pump or Multiple Daily Injections (MDI)**		
Pump	106	44.54%
MDI	132	55.46%
**Insurance**		
Medicaid	111	46.64%
Private	127	53.36%
**Type of Diabetes**		
1	228	95.79%
2	10	4.22%
**Race**		
Black	81	34.03%
White	146	61.34%
Hispanic	6	2.52%
Other	5	2.11%

### Hemoglobin A1c before the quarantine period

First, Hemoglobin A1c values over the pre-pandemic year, from May 2019April 2020, for each patient were analyzed (see Table2). Before the quarantine period, the average A1c value of patients in our clinic was 9.2%, with a range of 5.815%. The median value was 8.9%. There was no significant difference observed by gender (*p*=0.4959), or type of diabetes (*p*=0.8398). Patients on MDI showed significantly higher mean A1c of 9.6%, compared to those using pumps, at 8.8% (*p*=0.0003). Those on Medicaid showed a significantly higher mean A1c over the previous year, at 9.8%, compared to those on private insurance, at 8.8% (*p*<0.0001). Black patients had a significantly higher mean A1c, at 10.0%, than white patients, at 8.8% (p<0.0001).

**Table 2:** Comparing Hemoglobin A1C values before and during the quarantine by patient characteristics and significance of change

		Hemoglobin A1c Before quarantine May 2019April 2020			Hemoglobin A1c During quarantine MayJuly 2020			Change in Hemoglobin A1c from before to during quarantine		
	N	Mean	SD	p	Mean	SD	p	Mean	SD	p
**Sex**				0.4959			0.9976			0.4346
Female	107	9.15	1.56		9.48	2.11		+0.33	1.63	
Male	131	9.30	1.83		9.48	1.97		+0.18	1.25	
**Pump or Multiple Daily Injections (MDI)**				**0.0003**			**0.0006**			0.5527
Pump	106	8.81	1.37		8.99	1.75		+0.18	1.26	
MDI	132	9.58	1.876		9.87	2.16		+0.29	1.57	
**Insurance**				**<0.0001**			**<0.0001**			**0.0137**
Medicaid	111	9.77	1.78		10.26	2.07		+0.49	1.56	
Private	127	8.77	1.51		8.80	1.73		+0.03	1.29	
**Type of Diabetes**				0.8398			0.7857			0.8946
1	227	9.23	1.69		9.46	1.98		+0.23	1.32	
2	10	9.37	2.15		9.74	3.15		+0.37	3.20	
**Race**				**<0.0001**			**<0.0001**			0.7277
Black	81	10.08	2.02		10.36	2.24		+0.28	1.70	
White	146	8.81	1.32		9.02	1.75		+0.21	1.25	

### Hemoglobin A1c during the quarantine period

Next, the A1c values from MayJuly 2020, called the quarantine period, were analyzed (see Table 2). During the quarantine period, the average A1c value of patients in our clinic was 9.5%. Similar to the pre-quarantine period, no significant difference in A1c was observed by sex (*p*=0.9976), or type of diabetes (*p*=0.7857) during this period. Patients using MDI showed significantly higher mean A1c, at 9.9%, compared to those using pumps, at 9.0% (*p*=0.0006), and those on Medicaid showed significantly higher mean A1c, at 10.3%, compared to those on private insurance, at 8.8% (*p*<0.0001). Black patients also had a significantly higher mean A1c, at 10.4%, than white patients, at 9.0%, (*p*<0.0001) during the quarantine period.

### Hemoglobin A1c change in different clinical groups

**Fig. 1:**
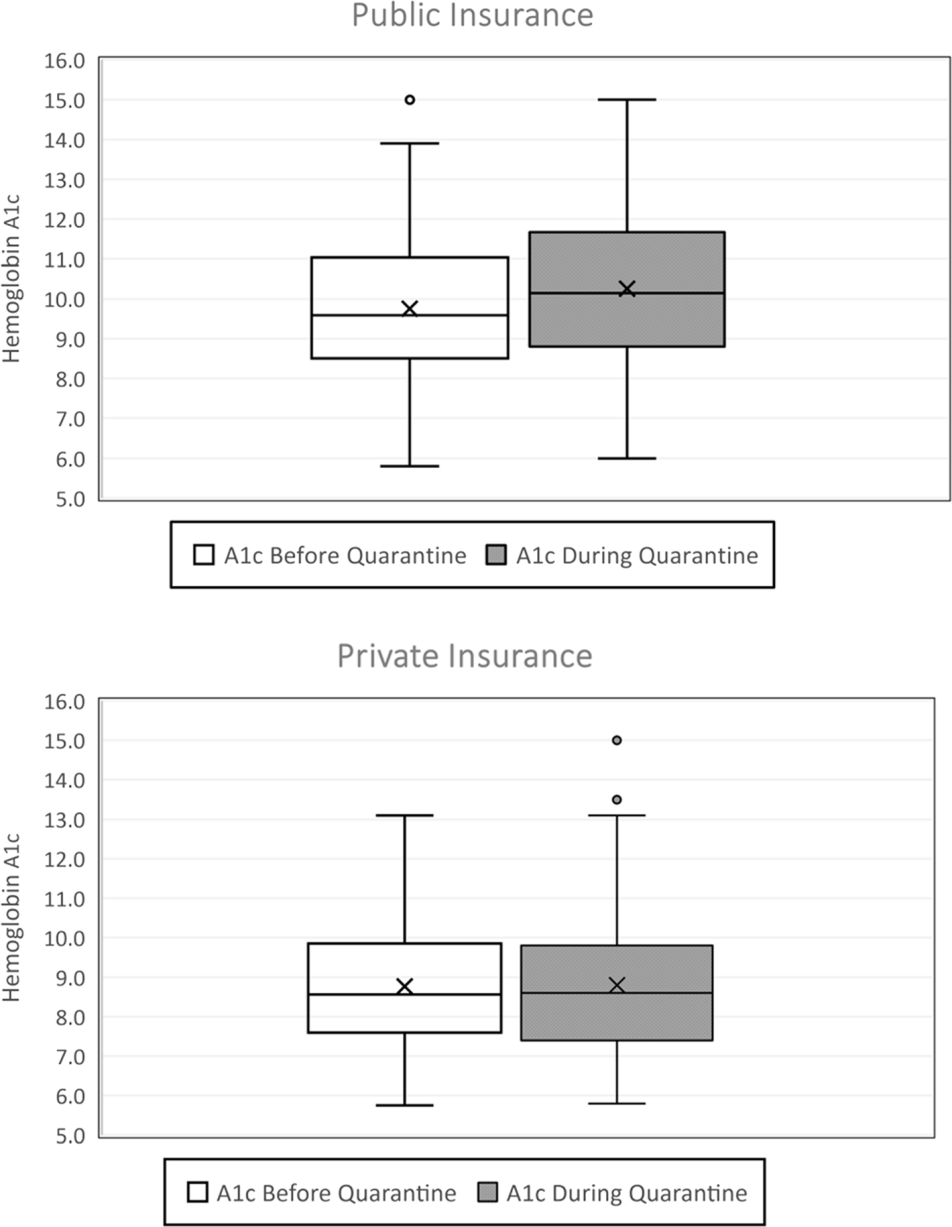
Hemoglobin A1c values before and during quarantine, compared by type of insurance

Next, we analyzed the change in A1c values from the pre-quarantine to quarantine period for different clinical groups (see Table 2). There was no significant difference in the average A1c change from the pre-quarantine period to the quarantine period in regards to sex (*p*=0.4346), mode of insulin administration (*p*=0.5527), type of diabetes (*p*=0.8946), or race (*p*=0.7277). However, there was significant difference by the type of insurance (*p*=0.0137). The average increase (0.49%) observed by Medicaid patients was significantly higher than that observed by private insurance patients (0.03%), see Fig.1.

### Hemoglobin A1c overall change from previous year to quarantine period

The average Hemoglobin A1c for our patients over the pre-quarantine period was 9.2%, and increased during the quarantine period to 9.5%. The average increase of 0.24% in A1c from pre-quarantine to quarantine period was statistically significant (*p*=0.0097). Seventeen percent of our patients were at the American Diabetes Associations goal of 7.5% or less before the pandemic and a similar amount (19%) were at goal during the quarantine period. Fifty-six percent of patients (*N*=132) had some increase in A1c level, while 44% of patients had a decrease in A1c level or no change. Of those 132 patients that experienced an increase, 30% of them had an increase of <0.5, and 70% had an increase of >0.5%. The percent of patients experiencing an increase in A1c is significantly higher than that would have been observed by chance variation (i.e. 5050% increase-decrease). The distribution of difference in A1c levels is fairly symmetric with about the same number of outliers on both ends indicating there are patients who experienced an unusually high increase in A1c level during pandemic compared to the rest of the patients and about the same number who experienced an unusually high decrease.

### Hemoglobin A1c direction of change (decrease or no change, increase of 0.10.4%, increase of >0.5%) in different clinical groups

Finally, we analyzed the direction of change: whether or not the patients A1c had a decrease or no change, increased by <0.5%, or increased by >0.5%, from the pre-quarantine to the quarantine period, according to patients characteristics (See Table3). In total, 44% of patients had a decrease or no change, 17% increased by <0.5, and 39% (*N*=92) increased by >0.5%. Of these 92 total patients that had the largest A1c increase of >0.5, 12% (*N*=11) of them were at an A1c goal of 7.5% or less before the quarantine period, and only one was still at goal during the quarantine period.

**Table 3:** Hemoglobin A1c Direction of Change In Different Clinical Groups

	Direction of Change of A1c						P
	Decrease or No Change		Increase of 0.10.4%		Increase of **>** 0.5%		
	N	%	N	%	N	%	
**Sex**							0.8904
Female	47	43.93%	17	15.89%	43	40.19%	
Male	59	45.04%	23	17.56%	49	37.40%	
**Pump or Multiple Daily Injections (MDI)**							0.1941
Pump	45	42.45%	23	21.70%	38	35.85%	
MDI	61	46.21%	17	12.88%	54	40.91%	
**Insurance**							**0.0079**
Medicaid	38	34.23%	20	18.02%	53	47.75%	
Private	68	53.54%	20	15.75%	39	30.71%	
**Type of Diabetes**							
1	101	44.49%	40	17.62%	86	37.89%	
2	5	50.00%	0	0.00%	5	50.00%	
**Race**							0.0752
Black	36	44.44%	8	9.88%	37	45.68%	
White	64	43.84%	30	20.55%	52	35.62%	

Among the different groups, there were no significant associations observed between the direction of change and sex of patient (*p*=0.8904), mode of insulin administration (*p*=0.1941), or race (*p*=0.0752). Association of type of diabetes and direction of change of A1c could not be analyzed because none of the Type 2 patients had an increase of 0.10.4%. Five patients had a decrease or no change, and the other five patients had an increase of >0.5%.

Significant association was observed, however, between the direction of change and the type of insurance. Forty-eight percent of Medicaid patients, versus 31% of private insurance patients, had an A1c increase of >0.5% (*p*=0.0079). On the other hand, 54% of private insurance patients experienced decrease or no change in HbA1c as compared to 34% of Medicaid patients (Table 3).

## Discussion

Our study shows that glycemic control in children with Type 1 Diabetes worsened during the initial quarantine period of the COVID-19 pandemic, with children on public insurance affected in greater proportion than those with private insurance. Before and during the quarantine, Medicaid patients had a higher A1c than private insurance patients, and Black patients had a higher A1c than white patients. The patients on MDI also had higher A1c then those on insulin pumps. Additionally, the average A1c increased significantly over time, in the whole group, from 9.2 to 9.5%. More than half of our patients had some increase in A1c during the quarantine period. Thirty-nine percent of our patients had an A1c increase of >0.5, and 24% had an increase of >1%. We also saw a statistically significant association between the type of insurance and the amount of change in A1c values. Medicaid patients experienced a significantly higher increase in A1c over the quarantine period at 0.49% as compared to private insurance patients at 0.03%. Forty-eight percent of Medicaid patients had an A1c increase of >0.5%, as compared to 31% of private insurance patients.

The pre-pandemic average A1c of patients (9.2%) in our study is very similar to the average A1c reported in larger studies. The T1D Exchange Registry reported an average A1c of 8.1% at 7years of age and a trend up to 9.2% by 19years of age in 2015 [5]. The SEARCH for Diabetes in Youth Study in 2019 looked at 1095 children with Type 1 Diabetes and reported the average A1c for their patients as 9.2% [6].

We found that patients on insulin pumps had an average A1c that was significantly lower than patients on MDI. The SEARCH for Diabetes in Youth Study in 2019 had similar findings, with an A1c of 8.9% in pump patients and 9.6% in those on MDI [6]. Johns et al. also found better glucose control with adolescents using pump therapy [7].

In our patient population, Black children had a higher A1c both before and during the quarantine than white children. This is similar to other studies that found that minority children often have a higher A1c than their white counterparts in the same area [7,10]. Redondo et al. reported a study showing that minority youth also have increased markers of poor long-term prognosis, such as high body mass index and/or hypertension, which increases risk of long-term health complications compared to white children [11].

Patients with public health insurance (Medicaid) had a significantly higher A1c both before and during the quarantine phase, and had a significantly higher increase in A1c during the quarantine phase, than those with private insurance. In 2014, Majidi et al. reported similar findings: youth with public insurance had a higher A1c than those with private insurance, but the effect was no longer seen once insulin regimen was controlled for [12]. Other studies have also shown that youth from lower-income families have worse glucose control [7, 10]. Secrest et al. looked at 317 children with Type 1 Diabetes who were followed to the age of 28years, and found that low-income status was inversely associated with A1c level which suggested that these individuals are at greater risk for complications such as autonomic neuropathy and lower-extremity arterial disease [13].

Access to good diabetes care can affect long-term outcomes in youth with Type 1 Diabetes, as shown by Valenzuela et al., who looked at 780 youth and found that those with low family income often face barriers to adequate healthcare, and that is associated with higher A1c levels [14]. Scott et al. report that adults with type 1 diabetes and low socioeconomic status have worse outcomes, possibly due to poor diabetes management [15], indicating that these factors starting in childhood have long-term impact. We believe that one barrier to care is lack of access to school nurses and trained school staff, who can provide oversight of diabetes management, which can lead to better blood glucose control.

Many studies have shown that school nurses can have a significant impact on improving diabetes care in children [1, 16,18]. When schools closed due to the quarantine, disadvantaged children were affected the most, due to lack of health care from school nurses and other factors [19]. Esposito et al. detailed the possible consequences of school closing, including deepening social and health inequities, and caution that the advantages of school closure need to be balanced with the adverse secondary effects [20]. In our study, we have shown worsening diabetes control in our population during the stay-at-home orders, with the most at-risk children, those on public insurance, affected the most. One possible cause of this worsening of control could be due to the school closures. In the event of future school closures, we will need to take additional steps to make sure that the children and families receive the help that they need. One of our local school districts has developed a possible solution. For children that chose to stay virtual for the 20202021 school year, the school nurse still called the families of the children with diabetes at mealtimes, to make sure they were checking the blood sugar at lunch and giving the proper insulin dosage, which seemed to help.

Another factor that is often overlooked in diabetes control is the impact by the family on children with type 1 diabetes. During the quarantine phase, the family dynamic shifted for many of our patients grandparents took on the role of diabetes caregiver, often with little or no training, or the young child was left in control of their own diabetes while the parent went to work and schools were closed. Studies have shown that parents with low health literacy have difficulty managing the complex insulin regimens [21]. In some of our patients families, the school nurse provided most of the diabetes care. With schools closing suddenly, there was little time to make sure that parents who needed extra help were given adequate training.

Many of our patients reported higher stress levels and anxiety during their clinic visits. Emotional climate at home can affect adherence to medical treatments [22], as can food insecurity [23], and we believe that these quarantine changes also affected our patients diabetes control, leading to higher A1c levels.

Limitations to the study include 30% of patients did not attend their scheduled clinic visit during the quarantine period, which may have limited the data collection. Even so, our results were statistically significant. We analyzed those patients who rescheduled, and compared their A1c over the previous year, to the most recent A1c once they came back to clinic in late 2020 or 2021, and they also had an average 0.3% increase in A1c, from 10.1% before the quarantine period to 10.4% afterwards. This is a similar increase to our study patients, so we do not think that it affected the outcome of this study. Another limitation was that 5% patients had only one prior A1c value in the previous year, so a clear trend could not be analyzed on those patients. Finally, our data is limited by not being able to include continuous glucose monitoring data, or more specifically Time-In-Range, since only 29% of our patients were wearing sensors during the quarantine phase. Many did not have a sensor, and others had one but were not wearing it.

## Conclusions

In summary, our study shows that the most at-risk population, Medicaid patients, had worse diabetes outcomes during the quarantine phase, which strengthens the conclusion that COVID has disproportionately affected minorities [24] and other vulnerable populations such as those with chronic illness [25]. It also shows that diabetes control worsened, even in those not infected with COVID-19, and strengthens the hypothesis that being in school helped control diabetes among children. Possible influential factors for worsening control include lack of routine, lack of school nurse oversight, family stressors, more snacking from staying home all day with little to do, and lack of exercise since everyone stayed indoors. Perhaps in the future, more resources could be offered to patients in lower socioeconomic groups during such a quarantine period, such as home visit nurses or extra virtual diabetes education.

## Data Availability

The dataset used for the current study are available from the corresponding author on reasonable request.

## Abbreviations

A1c: Hemoglobin A1c
MDI: Multiple Daily Injections
